# The personal and the public in residents’ burnout: a cross-sectional investigation

**DOI:** 10.1186/s13584-025-00742-z

**Published:** 2026-01-05

**Authors:** Yehonatan Sonvani, Shoval Madmon Sarikov, Tony Gutentag

**Affiliations:** 1 Gray Faculty of Medical & Health Sciences, School of Medicine, Tel Aviv University, Tel Aviv, 69978 Israel; 2 Gray Faculty of Medical & Health Sciences, Dental School, Tel Aviv University, Tel Aviv, 69978 Israel; 3 Gray Faculty of Medical & Health Sciences, Department of Medical Education, Tel Aviv University, Tel Aviv, 69978 Israel

**Keywords:** Burnout, Turnover, Satisfaction, Work-family conflict, Spillover

## Abstract

**Background:**

Medical residents are especially prone to burnout. Burnout has historically been viewed as a work-related condition. Burnout also occurs in life domains other than work, such as relationships and parenthood. The present investigation takes a holistic view of resident burnout, considering both public (i.e., work-related) and personal (i.e., couple, parental) burnout. The present investigation examines the interplay between resident burnout in different life domains, their antecedent (i.e., work-family conflict), and outcomes (i.e., job, couple, and parental satisfaction; turnover and breakup intentions).

**Methods:**

A pre-registered, cross-sectional study using questionnaires was conducted with 200 residents (*M* = 34.27 years, *SD* = 4.18; 63% female) in relationships and with children. We assessed work-related, couple, and parental burnout. The burnout antecedent was work-family conflict. The burnout outcomes included job, couple, and parental satisfaction, as well as turnover and breakup intentions.

**Results:**

As hypothesized, greater work-related burnout was associated with greater personal burnout. Work-family conflict was associated with higher levels of burnout, both work-related and personal. Higher work-related burnout was associated with lower job satisfaction and higher turnover intentions, showing domain-specificity. Higher couple burnout was associated with lower relationship satisfaction and higher breakup intentions, showing domain-specificity. Greater parental burnout was associated with lower parental satisfaction, as expected. However, this association was not domain-specific, contrary to the prediction: parental satisfaction was associated with all forms of burnout to the same extent (i.e., parental, couple, work-related).

**Conclusions:**

The findings shed light on the less-spoken, personal aspect of resident burnout. Burnout was domain-specific in some life domains (i.e., work, romantic relationship) but also spilled over from one life domain into another (i.e., parenthood). These findings underscore the importance of adopting a holistic bio-psycho-social perspective on residents’ well-being. Resident well-being is not only an individual matter but also a strategic priority for building a resilient, high-performing, and sustainable health workforce.

## Background

Burnout has historically been viewed as a work-related condition [1, 2]. Medicine is a profession highly prone to work-related burnout [3], with work-related burnout peaking during residency years [4]. Burnout also occurs in life domains other than work. Less is known about burnout in the personal domain, such as relationships [5] and parenthood [6]. The present investigation aims to take a holistic view of burnout, considering both work-related and personal burnout. We examine whether burnout is explained by the antecedent work-family conflict. Moreover, we examine whether burnout in different life domains explains outcomes (e.g., satisfaction) in their respective domain (but not in other domains), showing domain-specificity. Understanding how burnout intertwines across different life domains will provide a comprehensive bio-psycho-social perspective on residents’ well-being.

### Burnout

Burnout is typically considered a context-specific disorder [7]. Studies of burnout initially focused on the occupational context, which is still the most popularly researched domain. Work-related burnout is a state of exhaustion that occurs among employees, characterized by extreme tiredness, reduced ability to regulate cognitive and emotional processes, and mental distancing [8]. High levels of work-related burnout are present in medicine [3]. Residents are at an increased risk of burnout [4, 9]. This is because they are routinely challenged with high job demands, low individual autonomy, and job stress that interferes with their home life [10]. In the present investigation, we focused on residents, given their proneness to work-related burnout.

Burnout is not only an organizational phenomenon. Burnout has been increasingly considered in life domains beyond the occupational context, such as relationships and parenthood. Couple burnout is conceptualized as a syndrome of physical, emotional, and mental exhaustion arising in individuals who invested their sense of life meaning in the romantic relationship [5]. Parental burnout is an exhaustion syndrome in the parenthood domain, characterized by emotional exhaustion, emotional distancing from one’s children, feelings of being fed up with the parental role, and a sense of discrepancy between one’s current and previous parental self [6]. Burnout in one life domain might go hand in hand with, and even exacerbate, burnout in other life domains. Indeed, work-related burnout has shown a moderate positive association with couple burnout [5, 11], and a low to moderate positive association with parental burnout [11]. To the best of our knowledge, no research has shown the association among burnout in all three domains: work, couple, and parental, let alone in medicine. To explore the interplay of burnout across life domains, in the present investigation we focused on residents with a partner and children, examining their work-related and personal (e.g., couple and parental) burnout. We hypothesized that work-related and personal burnout would be positively associated yet distinct.

### Burnout antecedent

Work-family conflict is a form of inter-role conflict in which the general demands of-, time devoted to-, and strain created by the family interfere with performing work-related responsibilities [12]. It is one of the antecedents of burnout [13]. This conflict sparks an energy depletion process, whereby an employee’s sustained effort to meet demands may drain their energy backup [13]. Drained energy, in turn, might explain burnout in different life domains. Indeed, work-family conflict has been equally and positively associated with work-related and parental burnout [14]. We hypothesized that greater work-family conflict would be associated with higher levels of burnout, both work-related and personal.

### Work-related outcomes

Negative consequences of burnout for an organization are typically reflected in employee withdrawal, either mentally (e.g., satisfaction) or physically (e.g., turnover) [13]. Therefore, in the present investigation, we focused on two work-related outcomes: attitudinal symptoms of work-related burnout (i.e., job satisfaction) and a behavioral symptom of work-related burnout (i.e., turnover intentions) [15]. A burned-out worker will likely exhibit job satisfaction erosion and, ultimately, leave their job. Job satisfaction is the pleasurable emotional state resulting from the appraisal of one’s job as achieving or facilitating the achievement of one’s job values [16]. Burned-out physicians have reported being less satisfied with their jobs [17]. Accordingly, we hypothesized that greater work-related burnout would be associated with lower job satisfaction.

Turnover is the rate at which employees leave a position and are replaced. Physicians’ turnover intentions have been found to predict actual turnover [18]. Turnover results in direct costs to healthcare organizations associated with recruitment, as well as lost revenue during recruitment, onboarding, and the time it takes for a new physician to reach optimal efficiency [19]. Burned-out physicians have been found to exhibit more thoughts or intentions to quit their jobs [17]. Accordingly, we hypothesized that greater work-related burnout would be associated with higher turnover intentions.

### Personal outcomes

Regarding the consequences of personal burnout, we used the receptive outcomes of work-related burnout. Specifically, we focused on couple satisfaction, parental satisfaction, and breakup intentions (there is no parallel of turnover/breakup intentions with respect to parenthood). Couple satisfaction is defined as the perception of the degree to which the needs in a romantic relationship are met and satisfied [20]. Couple burnout has been associated with lower couple satisfaction [20]. Accordingly, we hypothesized that higher couple burnout would be associated with lower couple satisfaction. Parental satisfaction refers to being content in the parental role [21]. Greater parental burnout has been associated with lower parental satisfaction [21, 22]. Accordingly, we hypothesized that greater parental burnout would be associated with lower parental satisfaction. A breakup is the end of a romantic relationship. Couple burnout may lead to the breakup of the relationship [23], although we could not locate empirical papers showing this association. We nevertheless hypothesized that higher couple burnout would be associated with higher breakup intentions.

### Domain-specificity

Burnout has been associated with domain-congruent outcomes, showing domain-specificity. Work-related burnout has been uniquely associated with work-related outcomes (e.g., job satisfaction, turnover intention), and parental burnout has been uniquely associated with parental outcomes (e.g., parental satisfaction) [22]. Domain-incongruent outcomes have received less research attention. However, generally, burnout has not been associated with domain-incongruent outcomes. Work-related burnout has not been found to predict romantic relationship outcomes (i.e., couple satisfaction) [24]. Parental burnout was found to have little to no direct impact on work-related outcomes [7]. Accordingly, in the present investigation, we hypothesized that burnout would show domain-specificity: work-related burnout would be more strongly associated with work-related outcomes (compared to personal outcomes), while personal burnout would be more strongly associated with personal outcomes (compared to work-related outcomes).

### The present investigation

The present investigation explored burnout in different life domains, their antecedent, and outcomes. We focused on residents, as they are prone to work-related burnout [3, 4]. We also targeted residents with a partner and children, assuming they are also prone to personal burnout. Drawing from prior research [5, 6, 25], our first hypothesis was that work-related and personal burnout would be positively associated yet distinct. Regarding the antecedent, our second hypothesis was that greater work-family conflict would be associated with higher levels of work-related and personal burnout [13]. Concerning outcomes, we expected that burnout outcomes would be domain-specific [7, 22, 24]. Specifically, our third hypothesis was that work-related burnout (compared to personal burnout) would be more strongly associated with work-related outcomes, namely lower job satisfaction and higher turnover intentions. Our fourth hypothesis was that personal burnout (compared to work-related burnout) would be more strongly associated with personal outcomes: specifically, higher couple burnout would be associated with lower couple satisfaction and higher breakup intentions, and greater parental burnout would be associated with lower parental satisfaction.

## Methods

This research was approved by Tel Aviv University’s Institutional Review Board (#0007115-1). We pre-registered our research hypotheses.

### Participants

We surveyed 200 participants (*M*_*age*_*=*34.27, *SD*_*age*_*=*4.18; 63% [127] female). All participants were residents (*M* = 2.58 years in residency, *SD* = 1.38) with a valid medical license, currently in their residency training (31 in gynecology, 29 in family medicine, 22 in pediatrics, 17 in internal medicine, 12 in oncology, 11 in dermatology, 9 in ophthalmology, 8 in neurology, 7 in surgery, 6 in emergency medicine, 6 in anesthesiology, 4 in orthopedics, 3 in urology, 3 in ear, nose, and throat, 3 in cardiology, 2 in plastic surgery, 18 in other training, 9 undisclosed). They were also all in romantic relationships (*M* = 9.47 years in relationship, *SD* = 4.84) and had children (*M* = 1.97 children, *SD* = 0.94). Data collection took place between February 22 and July 9, 2024, approximately 4 to 9 months after the outbreak of the October 7, 2023, war; 27% (54) participants were called up for reserve duty during the war. After completing the survey, they could choose to receive a voucher for a coffee and pastry ($6.64 USD) from a bakery chain with a store in every hospital. A power analysis using G*Power 3.0 [26] indicated that a sample of 193 is required to detect a small effect (*r* =.20; 1-β = 0.80; α = 0.05).

### Procedure

The survey was distributed to the entire sampling frame via the resident organization’s mailing list. A snowball sampling method was then used to achieve the desired sample size. Participants first read about the survey’s aim and anonymously gave their informed consent to participate. Next, they completed the scales in the following order: work-family conflict, work-related burnout, job satisfaction, turnover intentions, parental burnout, couple burnout, parental satisfaction, couple satisfaction, and breakup intentions. Finally, all participants reported their demographic information.

### Materials

#### Burnout

**Work-related burnout.** To assess work-related burnout, participants rated (1 = never, 5 = always) the 12-item Brief Burnout Assessment Tool [27] (e.g., “At work, I feel mentally exhausted”; α = 0.84).

**Couple burnout.** To assess couple burnout, participants rated (1 = never, 7 = always) the 10-item Couple Burnout Measure [28] (e.g., “When you think about your marriage/intimate relationship overall, how often have you felt: tired”; α = 0.81).

**Parental burnout.** To assess parental burnout, participants rated (0 = never, 6 = every day) the 5-item Brief Parental Burnout Assessment [6, 29] (e.g., “I have the sense that I’m really worn out as a parent”; α = 0.81).

#### Potential antecedent

**Work-family conflict.** To assess work-family conflict, participants rated their agreement (1 = strongly disagree, 7 = strongly agree) with the 5-item Family-Work Conflict scale [12] (e.g., “The demands of my family or spouse/partner interfere with work-related activities”; α = 0.86).

#### Potential outcomes

**Job satisfaction**. To assess job satisfaction, participants rated (1 = not at all, 7 = very much) two items: “I feel fairly well satisfied with my present job as a resident” and “All things considered (i.e., pay, promotion, supervisors, co-workers), how satisfied are you with your present job as a resident?” [12] (α =.84).

**Turnover intentions**. To assess work turnover intentions, participants rated (1 = not at all, 7 = very much) the item: “How often have you seriously considered quitting your present job as a resident?” [30].

**Couple satisfaction.** To assess couple satisfaction, participants rated (1 = not at all, 7 = very much) two items: “All in all, how satisfied would you say you are with your romantic relationship?” and “Taking everything together, how happy would you say you are in your romantic relationship?” [31] (α =.91).

**Breakup intentions**. To assess breakup intentions, participants rated (1 = not at all, 7 = very much) two items: “How often do you discuss, or have you considered divorce, separation, or terminating your relationship?” [32] and “If you had your life to live over, do you think you would engage in a relationship with the same person?” [33] (α =.80).

**Parental satisfaction**. To assess satisfaction with parenthood, participants rated (1 = not at all, 7 = very much) two items: *“*All in all, how satisfied would you say you are with your parenting*?”* [31] and “Taking everything together, how happy would you say you are from your role as a parent?” (α =.82).

### Statistical analysis

To compute the means and standard deviations and to assess the Pearson correlation between variables, we used SPSS 21.0. To test whether two dependent correlations sharing one variable are significantly different within the same sample, we used Lee and Preacher’s interactive calculator [34], which applies Fisher’s r-to-z transformation and Steiger’s [35] equations to compute a z-score and corresponding p-value for the difference. A priori levels of significance were 0.05, and hypothesis tests were 2-sided.

## Results

Descriptive statistics and key analyses appear in Table 1. Consistent with our first hypothesis, work-related burnout and personal (i.e., couple and parental) burnout were positively associated yet distinct, supporting the discriminant validity of the constructs [36]. The correlations between the different forms of burnout were similar in magnitude.

Consistent with our second hypothesis, work-family conflict was associated with higher levels of work-related and personal burnout. Work-family conflict was correlated with both parental and couple burnout to the same extent. Additionally, work-family conflict was correlated with both work-related and parental burnout to the same extent. However, work-family conflict was correlated more strongly with couple burnout than work-related burnout.

Consistent with our third hypothesis, greater work-related burnout was associated with lower job satisfaction and higher turnover intentions. These correlations were domain-specific: the correlations between work-related burnout and its work-related outcomes were significantly stronger than with personal burnout.

Consistent with our fourth hypothesis, greater couple burnout was associated with lower relationship satisfaction and higher breakup intentions. These correlations were domain-specific, as they were significantly stronger than with work-related or parental burnout. In addition, greater parental burnout was associated with lower parental satisfaction. However, contrary to our prediction, this correlation lacked domain specificity as parental satisfaction was associated to the same extent with parental, couple, and work-related burnout.

**Table 1:** Means, standard Deviations, pearson correlations [CI_95%_], and significance tests of burnout with an antecedent and outcomes (N = 200)

	M	SD	Correlations [CI_95%_]			Significance test		
			*r* _Work−related burnout_	*r* _Couple burnout_	*r* _Parental burnout_	Work-related vs. couple burnout	Work-related vs. parental burnout	Parental vs. couple burnout
Work-related burnout	2.52	0.53	1			-	-	*Z* = 0.65,
								*p* =.512
Couple burnout	2.90	1.24	.31* [.18,.43]	1		-	*Z* = 1.82,	-
							*p* =.069	
Parental burnout	2.11	1.19	.37* [.25,.49]	.47* [.36,.57]	1	*Z* = 1.16,	-	-
						*p* =.246		
Antecedent								
Work-family conflict	3.83	1.39	.20* [.06,.33]	.41* [.28,.52]	.35* [.22,.46]	*Z* = 2.27,	*Z* = 1.59,	*Z* = 0.67,
						*p* =.023	*p* =.112	*p* =.500
Outcomes								
Job satisfaction	4.21	1.29	− .46* [−.56,−.35]	− .06 [−.20,.07]	− .12 [−.25,.02]	*Z* = 4.19,	*Z* = 3.57,	*-*
						*p* <.001	*p* <.001	
Turnover intentions	2.91	1.53	.44* [.32,.54]	.18* [.05,.31]	.14* [.01,.28]	*Z* = 2.83,	*Z* = 3.24,	*-*
						*p* =.005	*p* =.001	
Couple satisfaction	4.98	1.36	− .14* [−.27,−.01]	− .63* [−.70,−.54]	− .30* [−.42,−.17]	*Z* = 5.83,	*-*	*Z* = 4.11,
						*p* <.001		*p* <.001
Breakup intentions	2.50	1.59	.08 [−.06,.22]	.67* [.59,.74]	.31* [.17,.43]	*Z* = 7.16,	*-*	*Z* = 4.65,
						*p* <.001		*p* <.001
Parental satisfaction	5.03	1.19	− .24* [−.36,−.10]	− .35* [−.47,−.22]	− .38* [−.49,−.26]	*-*	*Z* = 1.51,	*Z* = 0.33,
							*p* =.132	*p* =.739

Note. *p <.05

Results unchanged after controlling for age, sex, number of children, percentage of children under 6 years old, duration of the relationship with the partner, and whether the resident was called up for reserve duty during the war (Δ = 0-|0.06|). Additionally, these demographics were mostly unrelated to the three types of burnout (i.e., work-related, couple, parental), with one exception: work-related burnout was correlated with the number of children, *r*(197) = −.26, *p* <.001, such that residents with more children reported lower levels of work-related burnout.

## Discussion

In the present investigation, we explored burnout in its extreme: residents with a partner and children. We explored work-related and personal burnout, its antecedent and outcomes. Our first hypothesis, that work-related and personal burnout are positively associated yet distinct, was supported. This finding aligns with existing research showing positive associations between work-related and couple burnout [25, 28] and between work-related and parental burnout [11]. To the best of our knowledge, this is the first study to examine all three (i.e., work-related, couple, parental) in one research design. Residents who experience burnout at work are also more likely to experience burnout in their personal lives. Nevertheless, work-related burnout and personal burnout were distinct, reflecting different facets of the burnout phenomenon.

Our second hypothesis, that greater work-family conflict would be associated with greater burnout (both work-related and personal), was also supported. It seemed that when residents felt pulled between work and family, they became more burned out across all domains. This finding is aligned with reviews suggesting that work-family conflict is an antecedent of burnout due to energy depletion [13]. Additionally, it is in accordance with previous research showing that work-family conflict was equally associated with work-related and parental burnout [14]. Adding to prior research, the present investigation demonstrated that couple burnout was also explained by work-family conflict. While work-family conflict explained the two types of personal burnout (i.e., couple and parental) to the same extent, work-family conflict explained couple burnout more than work-related burnout. Couples experiencing burnout were especially vulnerable to work-family conflict.

Our third hypothesis was also supported, with greater work-related burnout associated with lower job satisfaction and higher turnover intentions, showing domain-specificity. This is consistent with previous research, which found that burned-out physicians tended to be less satisfied with their jobs and exhibited higher turnover intentions [17, 22]. The domain-specificity we found was also in line with previous research comparing work-related and parental burnout [7]. Therefore, work-related burnout outcomes appear to manifest specifically in the work domain.

Our fourth hypothesis concerned personal burnout. As expected, greater couple burnout was associated with lower relationship satisfaction and higher breakup intentions, showing domain-specificity. This finding aligns with research indicating that couple burnout was associated with lower couple satisfaction [20]. It is also in accordance with theory, suggesting that couple burnout may lead to the end of the relationship [23]. The domain-specificity found was also in accordance with previous research reporting that couple satisfaction was not predicted by work-related burnout [24]. Taken together, couple burnout outcomes seem to manifest in the couple domain, in particular.

In addition, greater parental burnout was associated with lower parental satisfaction, as expected, and consistent with previous research [21, 22]. However, contrary to our hypothesis, there was no domain-specificity. This contradicts previous research among the general population, which found that parental burnout had a unique effect on parental satisfaction [22]. We found that greater burnout (across all domains) was associated with lower parental satisfaction. Parenthood among residents appeared to be susceptible to pressures arising from work, romantic relationships, or parenthood. Another possible explanation for this could be that parenthood is such an intense experience that depleted resources—regardless of their source—explain low parental satisfaction. Further research is needed to directly examine these explanations.

Demographic variables (e.g., age, sex, number of children, relationship duration, and military draft status) were largely unrelated to burnout, and controlling for them did not alter the reported results. This suggests that the findings of the present investigation are robust and not confounded by demographic or contextual factors.

### Implications for health policy

The present investigation focused on burnout in its extreme, among residents with a partner and children. Burnout during this formative training stage may impair clinical performance, delay professional growth, and increase the risk of attrition from the health sector altogether—challenges with significant policy implications for workforce planning both in Israel and globally. Burnout might hamper resident training and, therefore, should be minimized.

The findings shed light on the association between public (i.e., work-related) and personal (i.e., couple, parental) burnout. This dual focus highlights the importance of viewing health workforce well-being as both a workplace and societal concern. Future research should examine whether the model we proposed is causal. To the extent that it is causal, it can be used to mitigate burnout. To the extent that work-related and personal burnout are mutually influencing factors, treating each can benefit the other. Work-family conflict was found to be an explanatory factor for both. Addressing this conflict might mitigate burnout across domains. Burnout was found to be both domain-specific (with respect to work-related and couple outcomes), while also spilling over from one life domain to the other (with respect to parental outcomes). This stresses the need to support residents holistically to deter burnout, by incorporating well-being into residency program design, providing institutional support systems, and developing policies that promote work-life balance for health workers in training. Given the international relevance of health worker burnout, these findings are applicable to health systems worldwide. Supporting trainees holistically is not only a matter of individual well-being but also a strategic priority for building a resilient, high-performing, and sustainable health workforce.

### Limitations and future research

This research has several limitations. First, this is a cross-sectional investigation. We considered work-family conflict as an antecedent of burnout, based on previous research [13]. Nonetheless, it is possible that greater burnout increases work-family conflict. Likewise, we expected greater burnout to decrease work, couple, and parental satisfaction and increase intentions to leave work or romantic relationships, based on previous reviews [13]. However, it is possible, for example, that people who tend to be less satisfied are more burned out. To test the directionality of these associations, future studies should use experimental or longitudinal designs. Second, given the difficulty of reaching residents and their limited time availability, this investigation focused on only one antecedent and several outcomes of burnout, but there may be others. Future research could examine the lack of support and job control as other antecedents, and occupational injuries and job performance as other outcomes [13]. Finally, the present investigation examined burnout among residents with a partner and children. In addition, this was done to study burnout in extreme circumstances, during war. Future research could explore burnout among people from the general population in less demanding occupations and during peaceful times to examine the extent to which our findings can be generalized. Alternatively, future research could also more thoroughly explore the effects of war (e.g., [37]) on burnout.

## Conclusions

The present investigation focused on burnout among residents with a partner and children. The findings shed light on the link between work-related burnout and personal burnout. Work-family conflict was found to be an explanatory factor for both. Burnout was both domain-specific (with respect to work-related and couple outcomes) while also spilling over from one domain to the other (with respect to parental outcomes). These findings stress the need to support residents holistically to deter burnout. Protecting the mental health of trainees is not only a matter of personal resilience but of strategic importance for sustaining high-quality care delivery.

## Data Availability

Research data will be available on request.
